# Association of Clinical and Radiological Features in Various Repetitive Stress Injuries

**DOI:** 10.7759/cureus.7692

**Published:** 2020-04-16

**Authors:** Nazia Azeem, Madiha Ariff

**Affiliations:** 1 Radiology, Sir Syed Hospital, Karachi, PAK; 2 Internal Medicine, Dow University of Health Sciences, Karachi, PAK

**Keywords:** repetitive stress injury, de quervain disease, tennis elbow, plantar fasciitis

## Abstract

Objectives

To assess the clinical and radiological features of repetitive stress injuries (RSIs), specifically de Quervain’s (DQ) disease, tennis elbow (TE), and plantar fasciitis (PF).

Methods

This was an observational study conducted for eight months (March 2019 to September 2019) at the Orthopedic Department in collaboration with the Radiology Department. Clinically diagnosed RSI patients from both genders, aged between 30 and 50 years, were included in the study. Clinical features that were considered include pain, swelling, and site of injury. Radiological findings from ultrasound, X-ray, and magnetic resonance imaging (MRI) were identified in all participants of the study. Data were analyzed using SPSS Version 21 (IBM Corp., Armonk, NY, USA). Data were represented as frequency and percentages.

Results

There were a total of 82 patients (40 males,42 females) with repetitive stress injury (RSI), with a mean age of 42.63±8.53 years. Of the 82 patients, 34 (41.4%) had DQ disease, 28 (34.1%) had TE, and 20 (24.4%) had PF. Pain and swelling were observed in all patients. In TE, the most affected site was the right lateral epicondyle process. The common radiological findings were hypoechoic fascia, increased fluid within the first extensor tendon compartment, cortical erosion, sclerosis in soft tissues, and calcification in soft tissues. Cozen’s test was positive in most patients. In DQ, the most affected sites were left and right radial styloid processes. The common radiological findings were hypoechoic fascia, increased fluid within the first extensor, cortical erosion, and periosteal reaction. Finkelstein’s test was also positive in most patients. In PT, the most affected site was the left heal plantar surface. The common radiological findings were hypoechoic fascia, increased thickness of fascia (>4.5 mm), increased fluid within the first extensor tendon compartment, cortical erosion, sclerosis in soft tissues, calcaneal spur, periosteal reaction, and calcification in soft tissues. Cozen’s and Finkelstein’s tests were also positive in most patients.

Conclusions

History, physical examination, laboratory findings, and imaging modalities all are important tools for the differential diagnosis of RSI. Our study results showed that if some clinical findings fail to diagnose any RSI, then ultrasound, X-ray, and magnetic resonance imaging (MRI) are the best and useful options before treatment.

## Introduction

“Repetitive stress injuries” (RSIs), also termed as “overuse syndromes”, include several occupational illnesses such as tennis elbow (TE), de Quervain's (DQ) disease, plantar fasciitis (PF), youth pitching elbow, runner's knee, and shin splints. Overuse syndrome is suggested if there is persistent or recurrent musculoskeletal pain with no history of trauma in the previous six weeks [1]. Overuse is a level of repetitive micro-trauma, where damage occurs at the cellular level, resulting from repetitive activities within the physiological range; however, when there is excessive repetitive stress, the musculotendinous unit becomes overloaded, which leads to overuse injury, which may affect the bone, muscles, tendons, and ligaments [2]. Risk factors include repetition, high force, awkward joint posture, direct pressure, vibration, and prolonged constrained posture [3,4]. The upper body parts are more affected, including the shoulder, elbow, wrist, and hands [5]. Individuals with diabetes, gout, hypothyroidism, rheumatoid arthritis, calcium pyrophosphate deposition, and tuberculosis are at high risk of overuse syndrome [6].

DQ disease, or DQ tenosynovitis, is painful stenosis of the tendon sheath of the abductor pollicis longus and extensor pollicis brevis tendons at the radial styloid process. This overuse injury is linked to golfing, piano playing, carrying a child in the arms for longer periods, and/or excessive use of mobile for text messaging [7-9]. DQ causes pain and swelling near the base of the thumb during pinching, grasping, and other movements involving the thumb and radial inclination of the wrist [10].

The PF mostly affects adults, causes pain and disability, and may restrict athletic activities, occupational duties, or routine tasks [11,12]. PF (also called plantar fasciopathy) is allied to overuse trauma, leading to micro-tears, and shows a degenerative nature rather than an inflammatory one. Several medical conditions are associated with PF, and the most prominent conditions are seronegative spondyloarthropathies and rheumatoid arthritis [13]. Multiple risk factors are regarding repetitive stress on the PF, such as improper footwear, increased body mass index, prolonged walking, or running [14]. Plantar calcaneal spurs (calcaneal enthesophytes) can also cause PF [15,16].

Lateral epicondylitis or TE is tendinosis characterized by chronic symptomatic degeneration of the tendon that affects tendon attachments of the extensor muscles of the forearm to the lateral epicondyle of the humerus [17]. Tennis playing, excessive typing, playing the piano, and doing other manual work cause an overuse of the wrist extensor muscles, with the extensor carpi radialis brevis (ECRB) being the most affected muscle [16,18]. Tendinosis shows a degenerative process instead of inflammation [19,20].

The clinical presentation, history, and physical examination are usually enough to diagnose RSIs [21]. However, sometimes there are conflicting clinical findings, and the clinical presentations may be mimic several disorders. Therefore, different imaging techniques are routinely used for confirming the diagnosis of these diseases and their important features, such as plain radiography, MRI, computed tomography (CT), and ultrasound [22,23]. This study was aimed to compare the clinical and radiological features of various RSIs, particularly TE, DQ disease, and PF. This study also aims to assess the clinical and radiological features in RSIs and to find out the association of clinical and radiological features in RSIs.

## Materials and methods

This is a cross-sectional observational study conducted through a convenient sampling technique carried out at the Orthopedic Department in collaboration with the Radiology Department at a private hospital in Karachi, Pakistan, for a duration of eight months (March 2019 to September 2019). The Institutional Review Board approval was obtained from the same private hospital, and informed consent was obtained from each patient under study protocol. A total of 82 patients, both males and females, aged between 30 and 50 years, were divided into three groups: group I had 28 patients with TE, group II had 34 patients with DQ disease, and group III had 20 patients with PF cases who were already clinically and radiologically diagnosed. Patients with an infection that is associated with osteomyelitis and cellulitis were excluded.

Ultrasound, X-ray, and MRI were performed by a senior radiologist according to the specialization in a certain area. Results were then interpreted and recorded carefully. Ultrasound examination was performed using a linear 5- to 17-MHz probe (Philips iU22, Seattle, WA, USA), and the maximum thickness of fascia, hypoechoic fascia, adjacent fluid collection, and subcutaneous edema was recorded. X-ray was performed using a 200-mA X-ray machine (Toshiba, Tokyo, Japan), and findings included calcaneal spur, healing, cortical erosion, periosteal reaction, sclerosis in soft tissues, and calcification in soft tissues. All patients underwent MRI for confirming the diagnosis using Cozen’s Test and Finkelstein’s tests by a limited protocol specially designed for the study and a closed 1.5-T magnet (Avanto, Siemens, Erlangen, Germany) using an extremity coil. All findings were analyzed using SPSS Version 21 (IBM Corp., Armonk, NY, USA). Quantitative variables are expressed as frequency and percentages. Chi-square test was utilized as a statistical test, and p-value of <0.05 was considered as a significant difference.

## Results

There were a total of 82 patients (40 males, 42 females ), with a mean age of 30 to 50 years. Clinical features observed during physical examination in this study included frequency and intensity of pain, which were insignificantly associated between groups (p>0.05), frequency and intensity of swelling, which was significantly associated between groups, and site of injury, which include left radial styloid process, right radial styloid process, left lateral epicondyle process, right lateral epicondyle process, left heal plantar surface, and right heal plantar surface; there was a significant association between the groups (p<0.001) (Table 1).

**Table 1:** Clinical features of patients with repetitive stress injuries

Variable	Group I (n=28), n (%)	Group II (n=34), n (%)	Group III (n=20), n (%)	p-Value
Pain				
Mild	4 (14.3)	4 (11.8)	12 (60.0)	>0.999
Moderate	16 (57.1)	20 (58.8)	6 (30.0)	
Severe	8 (28.6)	10 (29.4)	2 (10.0)	
Swelling				
Mild	26 (92.9)	34 (100.0)	10 (50.0)	<0.001
Moderate	2 (7.1)	0 (0.0)	10 (50.0)	
Site				
Left radial styloid process	6 (21.4)	10 (29.4)	2 (10.0)	<0.001
Right radial styloid process	2 (7.1)	14 (41.2)	0 (0.0)	
Left lateral epicondyle process	8 (28.6)	4 (11.8)	4 (20.0)	
Right lateral epicondyle process	12 (42.9)	0 (0.0)	0 (0.0)	
Left heal planter surface	0 (0.0)	4 (11.8)	10 (50.0)	
Right heal planter surface	0 (0.0)	2 (5.9)	4 (20.0)	

Ultrasound findings in all groups showed increased thickness of fascia (>4.5 mm), which was observed in groups II and III whereas it was absent in group I. In groups I, II, and III, the hypoechoic fascia was seen, with an insignificant association between the groups (p=0.167). The increased fluid within the first extensor tendon compartment was found in groups I and II, whereas it was absent in group III, with a significant association between groups (p<0.001) (Table 2). X-ray findings include the presence of a calcaneal spur, which was observed in groups II and III, whereas it was absent in group I, with a significant association between the groups (p<0.001). Cortical erosions seen in all three groups, with an insignificant association between the groups (p=0.136). The periosteal reaction was found in all three groups, with a significant association between the groups (p=0.046). Sclerosis in soft tissues was seen in all three groups, with an insignificant association between the groups (p=0.556). Calcification in soft tissues was seen in groups I and III, and was absent in group II, with a significant association between the groups (p<0.001) (Table 2). MRI was performed to confirm Cozen's test, which was positive in groups I and III but negative in group II, with a significant association between the groups (p<0.001). Finkelstein’s test was positive in groups I, II, and III, with a significant association between the groups (p<0.001) (Table 2).

**Table 2:** Radiological findings of patients with repetitive stress injuries MRI, magnetic resonance imaging

	Group I (n=28), n (%)	Group II (n=34), n (%)	Group III (n=20), n (%)	p-Value
Ultrasound findings				
Increase thickness of fascia (>4.5 mm)	0 (0.0)	2 (5.9)	4 (20.0)	0.029
Hypoechoic fascia	6 (21.4)	2 (5.9)	2 (10.0)	0.167
Increased fluid within the first extensor tendon compartment	2 (7.1)	14 (41.2)	0 (0.0)	<0.001
Subcutaneous edema	0 (0.0)	0 (0.0)	0 (0.0)	-
X-ray findings				
Calcaneal spur	0 (0.0)	2 (5.9)	12 (60.0)	<0.001
Heal	0 (0.0)	0 (0.0)	0 (0.0)	-
Cortical erosion	6 (21.4)	14 (41.2)	4 (20.0)	0.136
Periosteal reaction	4 (14.3)	12 (35.3)	2 (10.0)	0.046
Sclerosis in soft tissues	6 (21.4)	4 (11.8)	4 (20.0)	0.556
Calcification in soft tissues	12 (42.9)	0 (0.0)	4 (20.0)	<0.001
MRI				
Cozen’s test	16 (57.1)	0 (0.0)	2 (10.0)	<0.001
Finkelstein’s test	2 (7.1)	18 (52.9)	2 (10.0)	<0.001

## Discussion

RSIs consist of a range of painful conditions of the musculoskeletal system, usually due to overuse. This study was conducted to assess the various clinical and radiological findings in different RSI conditions, particularly TE, DQ disease, and PF. Most of the time, sportsman, athletes, or other office-working people are more prone to these type of injuries and may get repetitive micro-injuries during playing, improper wrist or planter movements, excessive exercise, continuous running, improper footwear, sitting in the same position for a long period, etc.; this can also affect those who are typist or computer professionals, those who are piano player, those who use excessive mobile for text messages, etc. In this study, clinically diagnosed RSI patients were tested with different imaging modalities to identify radiological features of the same diseases.

TE (lateral epicondylitis) is an overuse syndrome that is most common in the fourth decade of life and is the most common cause of elbow pain. It is mostly diagnosed with clinical examination, but when symptoms are not enough to make a clear diagnosis, imaging diagnostic modalities are used [23]. Clinical symptoms of TE in this study were pain and swelling, with most of the patients having mild pain and mild swelling; however, the most affected site was the lateral epicondyle process (Table 1). Our results are similar to those of other previous studies that reported that TE is a painful condition that starts with the swelling of the tendons that attach to the lateral epicondyle of the humerus and continues as tendinosis, which is related to a repetitive contraction of the ECRB [24]. Ultrasound results showed hypoechoic fascia and increased fluid within the first extensor tendon compartment (Table 2); X-ray results showed cortical erosion, periosteal reaction, sclerosis in soft tissues, and calcification in soft tissues (Table 2); and MRI showed positive Cozen’s test in several patients, but only two had a positive Finkelstein’s test (Table 2). Other similar studies also depicted that the general appearance of lateral epicondylitis is a focal hypoechoic area in the deep part of the tendon (46/72) and that MRI has high contrast resolution and is, thus, a sensitive test for detecting lateral epicondylitis. Furthermore, sonography of the common extensor origin confirms the clinical notion of lateral epicondylitis, excludes other causes of lateral elbow pain, and gives useful information about the location, extent, and severity of lateral epicondylitis [25].

DQ disease is a result of repetitive, forceful, and ergonomically stressful work; the diagnosis of DQ tenosynovitis is based on a patient’s medical history as well as physical examination. Most patients with DQ complain about pain in the wrist when using the thumb and weak handgrip [7]. This study also found that moderate pain and swelling were present in all patients with DQ, and the most affected sites were the left and right radial styloid processes (Table 2). Ultrasound results showed increased fluid within the first extensor tendon compartment, which was most common in all patients; however, increased thickness of fascia (>4.5 mm) and hypoechoic fascia were also present in DQ patients (Table 2). X-ray results showed cortical erosion and periosteal reaction as the most obvious finding, whereas calcaneal spur, sclerosis in soft tissues, and calcification in soft tissues were also evident. MRI showed only a few patients with positive Cozen’s and Finkelstein’s tests (Table 2). A previous study described that an ultrasound scan of the symptomatic tendon displays distension in the tendon sheath with a fluid-filled surrounding that appeared like a diffuse circumferential hypoechogenicity. In DQ, the affected tendons were inflamed and thickened, and showed a significantly increased thickness compared with normal subjects [7]. Several studies are evident that radiological modalities are reliable and sensitive methods for detecting tenosynovitis [7,26].

PF, a disorder usually prevalent in the adult population, causes pain and disability in affected patients. Imaging is of great help for achieving a correct diagnosis, prompting appropriate treatment and aiding in the determination of prognosis [22]. The proximal third of the central bundle of the PF is classically involved; however, distal PF has recently been recognized as a cause of recalcitrant heel pain [27]. All patients with PF were found to have pain and swelling, with the most affected site being the left and right heal planter surface and left lateral epicondyle process (Table 2).

Another study showed that increased thickness of the PF measuring more than 4-5 mm within 5 mm of its calcaneal attachment is evident on lateral plain radiographs of patients with PF and represents a reliable sign of PF [28]. A recent study revealed that in the case of PF, MRI can assess thickening, signal changes, and edema of adjacent soft tissues. Radiographic findings of PF showed PF thickening, cortical irregularities, and abnormality in the fat pad located deep below the PF. Plantar fibromatosis becomes apparent and demarcated and PF tears present with partial or complete fiber disruption on both ultrasound and MRI [22]. We observe that the imaging techniques are more powerful in diagnosing RSI than just that physical examination and history; the studies also recommend the use of X-ray, ultrasound, and MRI to get accurate and timely results and gain detailed information regarding the exact pathology of pain [29,30].

## Conclusions

In light of the findings of this study, physical examination has its due importance in identifying the etiology of diseases, but for musculoskeletal disorders, many a time symptoms are not clear and require imaging methods to find the root cause of pain and swelling. We studied several RSIs and assessed their clinical and radiological findings; the results are in comparison with other previous studies, and the radiological findings are recommended before treatment selections.
